# Signals from the Niche: Insights into the Role of IGF-1 and IL-6 in Modulating Skeletal Muscle Fibrosis

**DOI:** 10.3390/cells8030232

**Published:** 2019-03-11

**Authors:** Laura Forcina, Carmen Miano, Bianca Maria Scicchitano, Antonio Musarò

**Affiliations:** 1 DAHFMO-Unit of Histology and Medical Embryology, Sapienza University of Rome, Laboratory Affiliated to Istituto Pasteur Italia—Fondazione Cenci Bolognetti, Via A. Scarpa, 14, 00161 Rome, Italy; laura.forcina@uniroma1.it (L.F.); carmen.miano@uniroma1.it (C.M.); 2 Istituto di Istologia ed Embriologia, Università Cattolica del Sacro Cuore, Fondazione Policlinico Universitario A. Gemelli IRCCS, 00168 Rome, Italy; biancamaria.scicchitano@unicatt.it

**Keywords:** skeletal muscle, fibrosis, muscle regeneration, Duchenne muscular dystrophy, aging, Interleukin-6, insulin-like growth factor 1

## Abstract

Muscle regeneration, characterized by the activation and proliferation of satellite cells and other precursors, is accompanied by an inflammatory response and the remodeling of the extracellular matrix (ECM), necessary to remove cellular debris and to mechanically support newly generated myofibers and activated satellite cells. Muscle repair can be considered concluded when the tissue architecture, vascularization, and innervation have been restored. Alterations in these connected mechanisms can impair muscle regeneration, leading to the replacement of functional muscle tissue with a fibrotic scar. In the present review, we will discuss the cellular mediators of fibrosis and how the altered expression and secretion of soluble mediators, such as IL-6 and IGF-1, can modulate regulatory networks involved in the altered regeneration and fibrosis during aging and diseases.

## 1. Introduction

Skeletal muscle fibrosis is a clinically relevant event that occurs under different physiopathologic conditions, such as aging and muscle diseases, or as a secondary outcome of traumatic events [1,2,3]. The deposition of fibrotic tissue is generally due to the impaired muscle regeneration, associated to an altered activity and balance of different cell components in skeletal muscle tissue [3,4]. The intimate relation between myogenic and non-myogenic progenitors, together with the mechanical connections and biochemical communication between resident cell populations, constitute a network of signaling pathways necessary to maintain muscle homeostasis and/or to guide an efficient regenerative process [5,6,7].

Mounting evidence indicates that multiple factors contribute to the alteration of tissue homeostasis, leading to the loss of the regenerative capacity of skeletal muscle and, thus, to fibrotic events [3,4]. The altered expression and secretion of soluble mediators, including cytokines and growth factors, can impinge cell–cell communication, affecting their physiological activity. In addition to the pivotal role exerted by satellite cells (SCs) and the activity of immune cells, other precursors and stem cell populations, either residing within the muscle or be recruited via the circulation in response to injury, can contribute to muscle regeneration. Among these, muscle-resident non-myogenic cells, such as fibro-adipogenic progenitors (FAPs), are determinant components of muscle niches contributing to the maintenance as well as to the alteration of the homeostatic environment upon physiologic or pathologic conditions [8,9]. FAPs are a source of a suite of soluble factors, including Interleukin-6 (IL-6) and the Insulin-like growth factor-1 (IGF-1), supporting satellite cell proliferation and differentiation [8,10]. In contrast, under pathologic conditions (i.e., muscular dystrophies) or during aging, the loss of homeostatic signaling can lead to SC alteration and to the fibro-adipogenic differentiation of FAPs [11,12]. A wealth of works supported the critical role of IGF-1 and IL-6 in muscle regeneration and disease. IGF-1 and controlled levels of IL-6 exert a pro-myogenic activity, whereas increased plasma levels of IL-6 have a detrimental impact on muscle homeostasis [13,14,15,16,17]. In this review, we focus on the main cellular and molecular players regulating the balance between muscle regeneration and fibrosis. In particular, we report insights into the role of IL-6 and IGF-1 in modulating the regulatory networks involved in the altered regeneration and fibrosis during aging and diseases.

## 2. Cellular Mediators of Regenerative Fibrogenesis and Fibrosis

**Figure 1:**
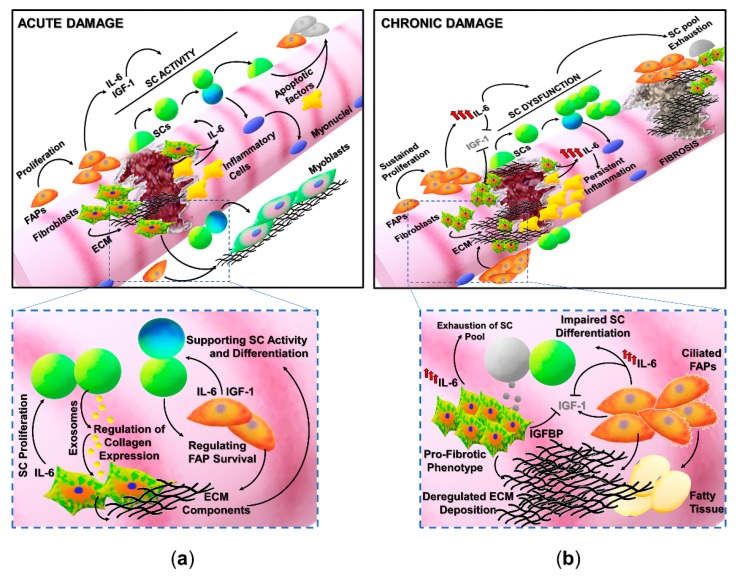
Regenerative fibrogenesis versus fibrosis: The occurrence of muscle fibrosis can be considered as the deregulation of events physiologically required to repristinate tissue homeostasis. (**a**) Fibrogenic pathways contribute to muscle healing, being involved in the adaptive response to acute damage. After muscle injury, the tightly regulated activation and proliferation of satellite cells (SCs), fibro-adipogenic progenitors (FAPs) (orange cells), fibroblasts (reported as green/orange cells), and inflammatory populations (yellow cells) are required for the efficient tissue repair. SCs, retaining stem-like properties, can undergo asymmetric division, giving rise to a daughter cell undertaking the myogenic program (blue cell) and to a cell able to regain the quiescent state (green cell) and contributing to the replenishment of the stem cell pool. Inflammatory cells and non-myogenic progenitors (FAPs) are involved in the removal of cell debris and the release of soluble mediators, like IL-6 and IGF-1, stimulating stem cell activity. The regenerative process is accompanied by the enhanced deposition and remodeling of the extracellular matrix (ECM), necessary to mechanically support newly generated myofibers and activated SCs. (**b**) Chronic degenerative stimuli can induce the alteration of interconnected mechanisms regulating cell populations in muscle niches. Indeed, cell populations involved in the physiologic response to muscle damage are the same players in the shift between the regeneration of functional tissue and the deposition of a fibrotic scar. FAPs and fibroblasts, which are a source of elevated levels of IL-6, can undergo deregulated proliferation, prevailing on SCs and driving the excessive deposition of ECM components. Moreover, elevated levels of IL-6 can induce a sustained proliferation of SCs and can impinge their myogenic differentiation. These alterations result in the production of fibrotic tissue at the expense of regenerative myogenesis. FAPs: Fibro-adipogenic progenitors; ECM: Extracellular matrix; SCs: Satellite cells; IGFBP: IGF binding protein.

Muscle regeneration is a homeostatic process in which the different phases involved in muscle healing, namely inflammation, satellite cells activation, remodeling, and maturation, must be finely regulated. Of note, satellite cells represent the main player in muscle regeneration; nevertheless, their activity can be modulated by different cell populations and precursors (Figure 1) [18,19,20,21,22].

Satellite cells, described by Mauro and Katz and identified as CD34^pos^/α7-integrin^pos^/Sca1^neg^/CD45^neg^/CD31^neg^ quiescent stem cells residing in a specialized niche, represent the myogenic stem cell pool responsible for the formation of new myofibers and for the repair of damaged muscle (Figure 1a) [23,24]. SC activity is known to be influenced by signals derived from the surrounding environment and by interactions with other cellular components of muscle niche [6,7,22]. In particular fibro-adipogenic progenitor cells (FAPs), identified as CD34^pos^/α7-integrin^neg^/Sca1^pos^/CD45^neg^/CD31^neg^ cells, expressing cell-surface platelet-derived growth factor receptor alpha (PDGFRα) are non-myogenic interstitial progenitors involved in the establishment of a dynamic environment supporting muscle regeneration, satellite cells proliferation, and differentiation [8,9,25,26].

The contribution of FAPs as regulators of muscle niches has been proposed, and the functional ratio between FAPs and SCs appeared as a prerequisite to guarantee muscle homeostasis [8,27,28].

Indeed, satellite cells and FAPs present a reciprocal regulation in muscle tissue, and their interactions are thought to be critical for an efficient regenerative process. It has been suggested that the reduction in the percentage of FAPs in aged muscles might result in an alteration in muscle homeostasis [11], whereas the presence of a significant number of FAPs, expressing high levels of IGF-1 and follistatin, in the extraocular muscles (EOMs) might contribute to the creation of a qualitative environment for SC activity, protecting the EOMs from age- and disease-related changes [11].

FAPs, which are normally quiescent in healthy muscles, actively proliferate following tissue damage, secrete a suite of factors that stimulate myogenesis, and are also known to transiently produce ECM proteins necessary to support and guide regenerative myogenesis (Figure 1a). It has been reported that the administration of Nilotinib, a tyrosine kinase inhibitor targeting PDGFR and TGFβ pathways critical for FAPs activity, severely affects regeneration in a mouse model of muscle damage [29]. However, it is worthy to indicate that Nilotinib, the drug used in the study by Fiore et al. (2016), is a broad-spectrum tyrosine kinase inhibitor and most likely targeted multiple cell types. Although FAPs can play a critical role in SC behavior and muscle regeneration, their activity must be transiently and finely regulated. The excess of FAPs, resulting from a physiologic regeneration, is subject to clearance mechanisms mainly mediated by apoptotic stimuli derived from immune cells and satellite cells [25,30]. In contrast, in several pathologic conditions, satellite cells or other precursor cells are thought to be defective in the production of factors that control FAPs activity. The altered activity of FAPs in a deregulated muscle environment, characteristic of chronic diseases, can contribute to the extensive ECM deposition and fatty tissue accumulation (Figure 1b) [31]. An aberrant accumulation and differentiation of FAPs have been described in aged muscles and in several models of chronic, degenerative diseases including Duchenne muscular dystrophy (DMD) and Amyotrophic lateral sclerosis (ALS).

It has been reported that severely denervated muscles presented a consistent accumulation of FAPs promoting a pro-fibrotic and pro-atrophic action in skeletal muscle [8,31,32]. An elective mechanism underlining the altered phenotype of denervated muscle-derived FAPs is the persistent stimulation of an IL-6 signaling pathway, since an enhanced expression and activity of IL-6 has been observed in FAPs isolated from different models of denervation. Moreover, the inhibition of STAT3, the main mediator of the IL-6 pathway, in a murine model of ALS (SOD G39A mice) prevented muscle atrophy and fibrosis [32]. Accordingly, the correlation between FAPs and IL-6 was also highlighted in the murine model of Duchenne muscular dystrophy (mdx mouse), a genetic muscle disease characterized by the progressive degeneration of muscle tissue, by inefficient regeneration, and by fibrotic/fatty tissue deposition. The overexpression of circulating IL-6 in mdx dystrophic mice (mdx/IL-6 mice) induced a significant exacerbation of the dystrophic phenotype with the long-term exhaustion of the satellite cell pool and the accumulation of FAPs [33]. It has been proposed that deregulated FAPs not only can prevail on satellite cell activity and can alter muscle niche but have been also indicated as potential progenitors of myofibroblasts and adipocytes, contributing to fibrosis and adipose tissue accumulation [34,35]. Interestingly, it has been recently reported that FAPs presenting primary cilia are involved in the deposition of fatty tissue in skeletal muscle upon injury or in cases of muscular dystrophy (Figure 1b, lower panel) [35,36]. Nonmotile cilia are responsible for the transduction of specific signals as the ciliary-Hedgehog (Hh) pathway that has been involved in both muscle repair and adipogenic differentiation of FAPs [35,36,37,38]. In contrast, the absence of cilia in FAPs stimulated myogenesis after injury and prevented adipogenesis in dystrophic muscles, highlighting a novel potential mechanism contributing to the regulation of muscle regeneration.

Although FAPs are critical players for muscle function and SC activity, the mechanism determining deregulated FAP activation remains to be fully elucidated, and the contribution of FAPs to skeletal muscle alteration is still debated.

### Reciprocal Regulation of Fibroblasts and Satellite Cells Activity

The production and secretion of extracellular matrix (ECM) components mainly depends on fibroblasts residing in muscle interstitium. The quality and abundance of ECM proteins are known to influence stem cell quiescence and activation within the muscle niche. However, different findings indicated that ECM deposition can be directly or indirectly regulated by other cell types as myogenic stem cells and progenitors (Figure 1) [39]. Satellite cells and fibroblasts are in close relation in regenerating muscles, since the remodeling of connective tissue is crucial to guarantee the mechanical support and biochemical signals necessary to activated stem cells. The specific role of fibroblasts during muscle regeneration has been poorly investigated because of the lack of specific markers. Nevertheless, a pioneer study, although not conclusive, suggests that the proliferation of connective tissue fibroblasts, identified by Tcf4 expression, strictly accompanies the expansion of activated Pax7^pos^ SCs after injury [40]. Although Tcf4 is also expressed by myoblasts and other cell types in adult skeletal muscle, these interstitial Tcf4^pos^ cells have been shown to express other markers of fibroblasts as PDGFRα and αSMA but not markers of myoblasts and macrophages as Pax7, MyoD, and F4/80. Since fibroblasts were found in close association with satellite cells and regenerating fibers, it is likely that their interactions play a role in the regulation of muscle repair. To support this hypothesis Murphy and colleagues performed a genetic manipulation of Tcf4^pos^ fibroblasts in injured skeletal muscle, observing a significant alteration in satellite cell behavior during regeneration. In particular, the dramatic reduction of fibroblasts in regenerating muscles led to a precocious SC differentiation impinging their expansion [40]. These data are in accordance with evidence demonstrating the involvement of fibroblasts not only in the deposition of ECM but also as a source of soluble factors that might regulate myogenic cells activity. For instance, fibroblasts are known to secrete growth factors, lipid mediators and cytokines as IL-6, involved in the regulation of SC proliferation and muscle metabolism [41]. However, the deregulated activity of fibroblasts under pathologic conditions can contribute to the alteration of homeostatic responses in skeletal muscle (Figure 1b, lower panel). It has been reported that IL-6 can promote the expression of collagen in human dermal fibroblasts via Gremlin1, a BMP inhibitor with pro-fibrotic actions, able to enhance TGFβ signaling pathway [42]. The reciprocal interaction between fibroblasts and muscle stem cells has been also elucidated by Fry and colleagues that reported how SCs can directly regulate ECM production by fibrogenic cells. In particular, it has been described that satellite cells can secrete exosomes containing a specific microRNA, the myoMiR-206, with important implications in muscle regeneration [43,44]. SC-derived exosomes have been shown to regulate collagen synthesis through the miR-206-mediated inhibition of Rrbp1, a master regulator of collagens expression [39]. These studies highlighted a potential mechanism by which SCs can actively modulate the local environment by influencing the biosynthesis of ECM components, further avoiding an excessive deposition of collagen during muscle adaptive responses (Figure 1b, lower panel).

If fibroblasts and secreted matricellular proteins have been shown to positively regulate physiologic muscle regeneration, it has also been reported that alterations in this compartment contribute to the loss of the muscle regenerative capacity during aging and disease. Changes in fibroblast features and ECM properties have been detected in aged muscles [2]. In particular, aged fibroblasts showed a contracted phenotype with nuclear deformation which is known to participate in the alteration of gene expression [2,45]. In accordance, a recent work by Stearns-Rider and colleagues showed that fibroblasts from aged muscles display a different expression of the collagen genes, with an increased expression of metalloproteinase inhibitors compared with ones isolated from young tissue. The altered composition of ECM and the increased stiffness associated with muscle aging can, in turn, induce a pathologic phenotype in fibroblasts, stimulating mechano-transductive signaling as the YAP/TAZ pathway, and can promote the fibrogenic differentiation of muscle stem cells [2].

The dysregulation of fibroblast activity and excessive ECM production have also been observed in chronic degenerative disorders as Duchenne muscular dystrophy (DMD). It has been proposed that the absence of the dystrophin protein may alter the structural and mechanical properties associated with fibroblasts. In addition, signals deriving from the altered milieu in dystrophic muscles can also contribute to the deregulated behavior of dystrophin-deficient fibroblasts. In fact, although the precise contribution of intrinsic and extrinsic alterations in the regulation of fibroblast activity has to be fully defined, it has been described that this cell population shows a pro-fibrotic phenotype in DMD (Figure 1b, lower panel) [46]. In particular, different studies from Zanotti and colleagues reported that primary fibroblasts derived from DMD patients showed a deregulated phenotype characterized by increased proliferation, altered collagen production, and a marked resistance to apoptosis [46,47]. Moreover, in vitro studies reported that DMD fibroblasts or their conditioned medium can impair the growth of dystrophic myoblasts. The putative negative impact of DMD fibroblasts on SC activity has been also related to the increased expression and secretion of the IGF-1 binding protein 5 (IGFBP-5), which might interfere with the pro-myogenic activity of IGF-1, since the neutralization of IGFBP-5 was able to revert the inhibitory effect of the DMD-fibroblast conditioned medium (Figure 1b, lower panel) [48].

Collectively these studies suggested that the enhanced fibroblast proliferation and ECM deposition, together with the altered expression of metalloproteinases and their inhibitors, can influence the activity of the muscle stem cell reservoir that could, in turn, affect their own behavior, fostering a fibrotic degenerative loop in dystrophic muscles. To support this hypothesis, when satellite cells were depleted from healthy overloaded muscles, an extensive ECM deposition and Tcf4^pos^ cell proliferation occurred, supporting the role of satellite cells in regulating fibroblast activity [39]. These evidences are coherent with a model in which the alteration of muscle fibroblasts and consequent modification of ECM properties can negatively influence satellite cell activity and fate. On the other hand, the progressive exhaustion of the resident stem cell pool in dystrophic muscles, due to intrinsic SC alterations and continuous cycles of degeneration and regeneration of myofibers, might allow fibroblasts to prevail on regenerative processes contributing to muscle fibrosis (Figure 1b).

## 3. Pro- and Anti-Fibrotic Factors Influencing the Balance between Regeneration and Fibrosis

The activation of fibrogenic pathways represents an adaptive mechanism involved in tissue repair after injury.

A key regulator of both myogenesis and ECM remodeling is the Transforming growth factor β (TGFβ), a cytokine with multiple roles in muscle healing and fibrosis [49]. TGFβ is known to be released by damaged myofibers and acts as a chemotactic factor for hematopoietic cells during the inflammatory stage of muscle regeneration. In addition, TGFβ has been recognized as a pivotal mediator of ECM remodeling, being a potent inducer of matricellular protein secretion by fibroblasts and a negative modulator of metalloproteinases. The physiologic increase of ECM components, due to TGFβ stimulation, plays an important role in the recovery of muscle architecture during healing, and its ablation in injured muscles can impair regeneration [49,50]. In contrast, TGFβ overproduction, that occurs under pathologic conditions, like DMD, is known to induce the excessive deposition of collagens, contributing to muscle fibrosis [51,52]. The activation of the TGFβ pathway through Smad3 signaling can also influence the proliferation and differentiation of muscle stem cells, inhibiting the activation of myogenic regulatory factors [53,54,55,56]. Furthermore, it has been observed that myoblasts overexpressing TGFβ differentiate into myofibroblasts when transplanted in murine muscles [57].

Myostatin is another member of the TGFβ family involved in muscle fibrosis [58]. The action of myostatin is mediated by a TGFβ-like signaling, involving the activation of the Smad/p38 MAPK/Akt axis, and can stimulate skeletal muscle fibroblast proliferation and the secretion of ECM proteins [49,58,59]. It has been reported that the genetic ablation of myostatin reduced fibrotic tissue deposition, favoring muscle regeneration in injured muscles [59]. Myostatin has been correlated not only to fibroblast proliferation but also to the resistance of DMD fibroblasts to apoptosis. Of note, the inhibition of myostatin signaling in dystrophic muscles was sufficient to revert the fibrotic phenotype [60]. Moreover, this myokine has been recognized as an inhibitor of muscle development and growth, impinging muscle progenitor cell proliferation and differentiation [55,61,62]. Notably, the pro-fibrotic action of myostatin can be counteracted by decorin, a proteoglycan involved in collagen fibrillogenesis [63]. The administration of decorin has been shown to prevent myoblast trans-differentiation into myofibroblasts in vivo and has led to the improved recovery of skeletal muscles after injury [57,64]. The anti-fibrotic action of decorin can be related to the upregulation of another anti-fibrotic agent, follistatin, which is an inhibitor of myostatin [59]. Indeed, follistatin overexpression improved myogenic differentiation in vitro and muscle regeneration in vivo, reducing muscle fibrosis [65].

Another cytokine referred as a myokine is IL-6, which is known to play important roles in muscle regeneration, inflammation, and fibrosis. IL-6 is involved in the regulation of satellite cell activity promoting the proliferation of activated stem cells, whilst its overexpression can impair myogenic differentiation and the physiologic resolution of the inflammatory response, fostering pathogenic changes that result in muscle wasting and fibrosis. Of note, IL-6 has also been correlated to the inhibition of IGF-1 activity, an anabolic and pro-myogenic factor critical for skeletal muscle growth and regeneration [66]. Thus, molecular mediators in muscle milieu, like IL-6 and IGF-1, can play an important role in regulating the balance between muscle regeneration and degeneration. These niche factors, modulating tissue environment, can act by supporting reparative mechanisms in injured tissues or can impair, when deregulated, the physiologic response to damage, thus promoting fibrotic tissue deposition.

## 4. Interleukin-6: Spectrum of Pro-Fibrotic Actions

**Figure 2:**
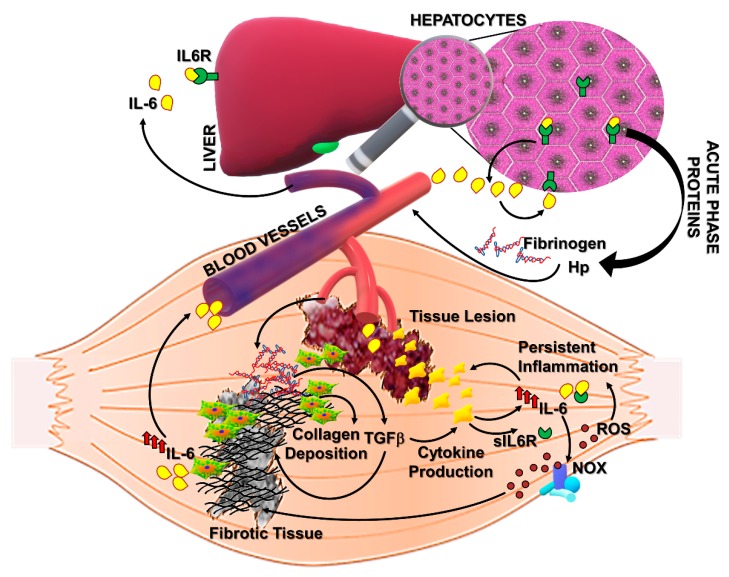
The mechanisms underlining the pro-fibrotic actions of IL-6 in skeletal muscle: Elevated levels of IL-6 are associated with inflammation-related pathologies in which the occurrence of fibrogenic events contributes to the severity of the disease. Hepatocytes, expressing the IL6R, are highly responsive to circulating IL-6, leading to an extensive production and secretion of acute phase proteins (APPs). The excessive accumulation of fibrinogen in damaged muscles can promote pathologic fibrogenesis. Fibroblasts (reported as green/orange cells) and infiltrated inflammatory cells (yellow cells) further contribute to the enhanced secretion and activity of IL-6, which can, in turn, affect the quality of inflammation and alter the muscle redox balance, fostering the extent of fibrotic tissue deposition. IL6R: IL-6 receptor alpha; sIL6R: soluble IL6R; Hp: Hepcidin; ROS: Reactive oxygen species; NOX: NAD(P)H oxidase complex.

IL-6 is a pleiotropic cytokine promptly produced by muscle and other tissues in response to physiopathological changes including physical exercise, infections, and injury [67]. The positive and negative roles of IL-6 in tissue homeostasis have been extensively studied and described, and it is generally accepted that the dual nature of its action is related to the activation of different signaling pathways [68,69]. The activation of the classical signaling is strictly dependent on the expression of the membrane bounded IL-6 receptor alpha (IL6R) in combination with the ubiquitous receptor gp130. Thus, the canonical signaling is physiologically restricted to those cells expressing IL6R and is responsible for the induction of anti-inflammatory and pro-regenerative pathways in skeletal muscle [15,70,71,72]. A soluble form of IL6R, sIL6R, is able to amplify the spectrum of action of IL-6, activating the so called trans-signaling with pro-inflammatory and pro-fibrotic implications (Figure 2) [42,73,74,75,76]. Of note, circulating IL-6 levels are undetectable under physiologic conditions but increase significantly in several diseases associated with inflammation and fibrosis [33,77,78,79].

### 4.1. Immune Response and Fibrosis: A Fatal Interplay

IL-6 is produced by a wide range of immune cells, which are, in turn, highly responsive to this cytokine. Moreover, the alternative signaling of IL-6, mediated by the sIL6R, can promote a chronic inflammatory status, influencing the quality of the immune response. In fact, IL-6 is known to affect the balance between leucocytic populations at the site of inflammation, inducing a shift from neutrophilic to macrophagic accumulation. A role for IL-6 in regulating T and B lymphocytes has been also reported, demonstrating that it can induce B and Th17 cells and can inhibit Treg differentiation, favoring the persistence of the inflammatory response [78,80,81,82].

According to the pivotal role of IL-6 in promoting the establishment of a chronic inflammatory environment, it has been described that IL-6 promotes a pro-fibrotic status in a mouse model of peritoneal inflammation by impairing the physiologic resolution of the inflammatory response through the promotion of the T helper 1 immune response [83]. Of note, IL-6 deficient mice were resistant to repeated inflammation-related fibrosis. In addition, it has been reported that the IL-6 signaling contributes to the development of fibrosis in the sclerodermatous chronic graft-versus-host disease (Scl-cGVHD), another model of immune-related disease characterized by excessive ECM deposition in the skin and visceral organs that impair tissue functionality [84]. In light of these and many other studies, IL-6 blockade clearly appears as a possible strategy to avoid fibrotic tissue degeneration. Pharmacological inhibitors of IL-6 signaling, which are already used in the clinic to treat chronic disorders like rheumatoid arthritis, have been shown to induce beneficial effects in a suite of experimental models of degenerative diseases [42,85,86,87]. For instance, Le Huu and colleagues demonstrated that the inhibition of IL-6 trans-signaling, through a neutralizing antibody direct against IL6R (MR16-1), reduced the extent of fibrosis in the skin, lungs, and liver from Scl-cGVHD mouse models by enhancing the number of T regulatory cells [84].

Chronic inflammatory changes and fibrogenic events also characterized the skeletal muscle environment in muscular dystrophies, where the constant induction and amplification of pro-inflammatory pathways interfered with regenerative signaling, contributing to muscle fibrosis. In this pathologic context, recent works highlighted the involvement of sustained IL-6 levels in inducing muscle wasting. It has been reported that elevated circulating levels of IL-6 are the determinant for the development of a severe phenotype in mdx mice, more closely approximating the human DMD pathology [33,77,79].

Of note, IL-6 is able to actively induce an extensive production and secretion of acute phase proteins (APPs), including fibrinogen (Figure 2) [88]. Fibrinogen can increase the permeability of blood vessels at the inflammatory lesion where it is enzymatically converted to polymeric fibrin [89,90,91]. The excessive fibrinogen accumulation and fibrin deposition have been reported in dystrophic muscles from DMD patients and mdx mice [92]. In particular, Vidal and colleagues observed a significant correlation between fibrin/ogen accumulation and fibrosis in DMD muscles, suggesting a role for fibrin/ogen in promoting pathologic fibrogenesis (Figure 2). IL-6, which is known to be significantly increased in DMD sera and muscles, can induce fibrinogen expression not only through the STAT3-mediated downstream pathway but also by directly interacting with responsive elements upstream the fibrinogen gene [93,94]. Fibrinogen expression can, in turn, induce collagen deposition by increasing TGFβ expression in mdx mice and stimulate a macrophage-dependent cytokine production (including IL-6), thus fostering a pro-fibrotic mechanism in DMD muscles (Figure 2). Other critical IL-6/STAT3-induced factors are haptoglobin (Hp) and Hepcidin, physiologically involved in the iron homeostasis, which are known to contribute, when deregulated, to the inflammation-related iron alteration [94,95,96]. Haptoglobin is a protective factor against tissue damage derived from free hemoglobin and consequent oxidative stress. In particular, Hp was reported as an anti-inflammatory factor inducing the expression of heme oxygenase (Ho-1) and IL-10. However, recent studies revealed that elevated levels of Hp can further induce IL-6 expression, possibly amplifying pathologic changes during persistent inflammation [94,97]. In fact, Hp has been recently proposed as a serum biomarker of diagnosis/prognosis for DMD [98]. Accordingly, circulating levels of both IL-6 and Hp strongly increased during chronic inflammatory conditions such as rheumatoid arthritis (RA) and DMD [98,99,100,101,102]. Although IL-6 fulfills beneficial roles in host defense and tissue repair, inducing the systemic acute phase response, it is plausible that a sustained stimulation of IL-6 signaling pathway could affect muscle homeostasis.

Of note, a phenotypical and functional improvement of dystrophic muscles has been observed under IL-6 trans-signaling inhibition [100]. Similar results have been recently obtained in dystrophin-/utrophin-deficient mice (dKO), a severe model of DMD [103]. Notably, Wada and colleagues revealed a reduced extent of muscle fibrosis and a significant down-modulation of pro-fibrotic factors, as periostin and Timp1, in adult dKO mice treated with neutralizing IL6R antibody compared to untreated mice [103].

Altogether these data indicated that IL-6 signaling can induce pro-fibrotic pathways in skeletal muscle and other tissues by promoting the establishment of a chronic pro-inflammatory milieu. Unresolved inflammation can, in turn, impede a physiologic regenerative process and drive fibrotic tissue deposition. Considering these evidences, IL-6 trans-signaling blockade appeared as a potential therapeutic approach with anti-fibrotic effects in treating inflammatory-related diseases.

### 4.2. Oxidative Stress

Another proposed mechanism underlining tissue fibrosis is the imbalance between reactive oxygen species (ROS) and the endogenous antioxidant defense. Mounting evidence supports the hypothesis of a “redox-fibrosis” occurring in aging- and inflammation-related diseases [104,105]. Excessive ROS production and markers of oxidative damage have been reported in several pathologic conditions as liver and lung fibrosis and in muscle wasting diseases like DMD. In particular, ROS production in skeletal muscle derives from a suite of cellular sources including immune cells, fibroblasts, endothelial cells, and myofibers which express the NAD(P)H oxidase complex (NOX) and are able to convert molecular oxygen to oxygen radicals (Figure 2). These highly reactive molecules are considered secondary mediators in different signaling involved in metabolic and regenerative pathways, and their production and persistence are finely regulated. In fact, the extent of ROS production is generally counterbalanced by antioxidant systems mainly regulated by Nrf2 transcription factor, which neutralizes free radicals. In particular, under oxidative conditions, Nrf2 is released by its regulator Keap1, acquiring the ability to translocate in the nuclear compartment and to regulate the gene expression of the main antioxidant enzymes, deputed to the neutralization of superoxide and its derivates [106,107,108]. Thus, the occurrence of oxidative stress can be promptly prevented by the maintenance of the homeostatic balance between oxidant and antioxidant mediators. However, ROS accumulation and antioxidant system impairment have been described under pathologic conditions leading to oxidative damage.

An altered redox status has been shown to be related to fibrosis persistence in aged mice. In particular, the enhanced expression of NOX and the depressed Nrf2-dependent antioxidant response contributed to the impaired capability of aged mice to resolve lung fibrosis, whereas targeting NOX4 was possible to reverse the fibrotic phenotype [109]. The involvement of NOX-related redox signaling in pro-fibrotic pathways has been also described in skeletal muscle tissue. NOX-dependent ROS production in muscle cells has been shown to be strictly involved in the pro-fibrotic action of Angiotensin II, an oligopeptide increased in fibrotic diseases which is able to induce the expression of a suite of ECM components including fibronectin and collagen-III (Figure 2) [110].

A significant increase in NOX2 expression and activity has been also described in DMD patients and dystrophic murine models. Of note, recent works revealed a pivotal role of IL-6 in inducing detrimental effects on the NOX2/Nrf2-dependent redox balance in dystrophic muscles. It has been demonstrated that enhanced serum levels of IL-6 in mdx mice (mdx/IL-6 model) dictated a perturbation of redox signaling cascades in dystrophin-deficient muscles even prior to the necrotic phase and during the progression of pathology [79]. Although dystrophic muscles are able to induce an active antioxidant response to counter the detrimental effects of ROS, the sustained expression of IL-6 in mdx/IL-6 mice stimulated ROS production by enhancing the NOX2 expression and impinged the Nfr2-dependent antioxidant defense, repressing the expression of both Nrf2 protein and Nrf2-dependent genes. [77,79]. A similar expression pattern of oxidant and antioxidant mediators was observed in DMD patients, characterized by high expression levels of IL-6 during the progression of pathology. Furthermore, data from our studies highlighted IL-6 as a possible link between inflammation and ROS production in dystrophic muscles [77,79]. In fact, sustained stimulation of IL-6 signaling not only impairs the endogenous antioxidant response in mdx muscles but also induces the upregulation of NOX2 and NF-kB, a critical mediator of the inflammatory response, fostering pathogenic mechanisms involved in muscle wasting and fibrosis (Figure 2) [33,77,108].

## 5. IGF-1 As a Modulatory Factor in Muscle Niche, Promoting Regenerative Events

An efficient muscle repair after injury strictly depends on the activation and completion of a proper regenerative program. Molecular mediators supporting satellite cell survival and differentiation could play a critical role in the promotion of a functional tissue recovery at the expense of fibrosis (Figure 1). IGF-1 is a growth factor with anabolic functions that has been implicated in muscle growth, hypertrophy, and regeneration [16,111,112,113,114]. IGF-1 is secreted by a suite of cell populations within the muscle niche, including satellite cells, myofibers, fibroblasts, and inflammatory cells, and likewise, cells are responsive to this peptide hormone [11,115,116,117]. In fact, the IGF1 receptor (IGF1R) is ubiquitous and activates molecular cascades critical for muscle homeostasis such as protein synthesis, muscle cell survival, and muscle activity [118,119]. After muscle injury, monocytes/macrophages recruited at the site of the lesion represent a primary source of IGF-1 [116,117]. The balance between different subpopulations of macrophages is essential for physiologic muscle regeneration after damage [18,21,120,121]. In particular, macrophages are a heterogenous cell population with a range of activation-related skills [122,123,124]. M1 macrophages, expressing surface markers, like CD68 and Ly6C, are considered pro-inflammatory cells participating in the early stages of muscle regeneration by removing cell debris and releasing cytokines able to amplify the inflammatory response and to activate muscle stem cells [122,125,126,127]. Alternative activated macrophages (M2), mainly characterized by markers as CD163, CD206, and CX3CR1, are involved in the resolution of inflammation and in the promotion of the myogenic program and ECM remodeling and play a role in the later stages of tissue repair [122,126,127]. In a recent work, Tonkin and colleagues reported that both Ly6C and CD206 positive macrophages contribute to IGF-1 production in injured muscles at the stage of the transition between inflammation and regeneration (day 5 after damage). The genetic ablation of IGF-1 in murine monocytes/macrophages (φIGF-1 CKO mouse) resulted in impaired muscle regeneration after an injury with a reduced size of regenerated myofibers, expanded interstitial spaces, and fatty tissue deposition [117]. In addition, φIGF-1 CKO mice presented an imbalance between the macrophagic subpopulation with a significant accumulation of Ly6C cells and a reduced presence of CD206 cells, indicating that the loss of IGF-1 expression could induce an alteration of the quality of the inflammatory response at the site of tissue lesion, affecting the switch from inflammation to regeneration. These data are consistent with a series of studies highlighting the role of IGF-1 in promoting muscle regeneration through the stimulation of myoblast proliferation and differentiation and by the modulation of muscle environment (Figure 1a). For instance, we demonstrated that the enhanced expression of mIGF-1, the local isoform of IGF-1, in skeletal muscle (MLC/mIGF-1 mouse) accelerates regenerative processes after muscle injury, creating a qualitative environment able to efficiently support a proper tissue repair. In particular, a murine muscle in which mIGF-1 was overexpressed rapidly regenerated after damage, reducing muscle fibrosis, and presented a modulated inflammatory milieu characterized by the reduced expression of pro-inflammatory cytokines and chemokines as TNFα, IL1β, and MIF [113].

The IGF-1 signaling pathway is known to be altered in skeletal muscles during aging when the decline of the regenerative capability of muscle tissue occurs [128]. In addition, reduced levels of circulating IGF-1 have been described in serum from elderly individuals with a positive correlation with the decline of protein synthesis and muscle mass and increased levels of myostatin and fatty tissue accumulation [129,130,131,132,133]. In contrast, the overexpression of the mIGF-1 transgene revealed a protective action of this specific isoform against skeletal muscle decline during senescence in mice [134]. Senescent transgenic mice (24 months of age) displayed attenuated signs of age-related changes in muscle phenotype compared to age-matched wild-type mice, retaining the ability to promote functional regenerative mechanisms [134]. Moreover, the stimulation of the Akt/mTOR axis downstream of IGF-1 signaling by Losartan, an inhibitor of Angiotensin II receptor (AT1), was able to counteract the occurrence of muscle atrophy due to disuse in aged mice [135]. Losartan-treated sarcopenic mice presented a reduced extent of fibrotic tissue deposition after injury, exhibiting a preserved muscle architecture upon damaging events compared to control mice [135].

The ability of IGF-1 to preserve the structural integrity of muscle tissue and to support regenerative mechanisms has been also elucidated under pathologic conditions as muscular dystrophies. The combination of IGF-1 overexpression and Losartan treatment significantly ameliorated the pathologic muscle phenotype in murine models of Lama2-related muscular dystrophy (MDC1A) by reducing inflammation and fibrosis and by preserving tissue structure and function [136]. The enhanced expression of mIGF-1 selectively in skeletal muscles of dystrophic mice influenced the offset between tissue degeneration and regeneration. In particular, it has been demonstrated that mdx/mIGF-1 mice not only presented a reduced myonecrosis of the diaphragm muscle, the most compromised muscular district in DMD, but also displayed functional hypertrophy. In addition, although IGF-1 has been recognized as a stimulator of fibroblast activity, mdx/mIGF-1 mice revealed a reduced extent of muscle fibrosis. The well-described pro-myogenic activity of IGF-1 in skeletal muscle can also contribute to the stabilization of dystrophic muscle phenotype by promoting survival pathways and molecular circuits, leading to a complete myogenic differentiation of regenerated fibers along with the maintenance of their terminally differentiated status [137,138]. Despite controversial indications about the anti-fibrotic role of IGF-1 in muscle tissue, a recent work highlighted a possible correlation between circulating levels of irisin, IGF-1 expression, and the improved muscle mass in human, or the amelioration of dystrophic muscle in mice [137,139,140,141]. Irisin is a myokine secreted in response to physical exercise able to induce muscle growth and to promote IGF-1 expression in human myocytes [140]. Reza and colleagues reported that irisin injection in mdx mice resulted in a significant reduction of muscle necrosis and fibrosis, enhancing the stability of the dystrophin-deficient sarcolemma [142]. Of note, the upregulation of IGF-1 was proposed as a possible molecular mechanism underlining irisin action and mediating its modulatory impact on dystrophic muscle.

## 6. Conclusions

The enhanced production and deposition of ECM components represent a critical stage of the adaptive response of muscle to tissue injury, directed to the generation of mechanical and biochemical support for regenerating fibers. Regenerative fibrogenesis is the result of a well-coordinated network of signals deriving from different cell contributors, and it is strictly required to efficiently repristinate the proper architecture of the functional muscle tissue. When the orchestrated pattern of fibrogenic stimuli is disturbed, as under physiopathologic conditions like aging and muscular dystrophies, tissue fibrosis occurs, contributing to the progressive impairment of muscle function. Thus, the study and identification of molecular and cellular mediators involved in the alteration of fibrogenic events appear of relevant clinical interest. Among the factors, IGF-1 and IL-6 can be considered as central mediators in the muscle niche, influencing the balance between muscle regeneration and fibrosis. In particular, IGF-1 can favor regenerative myogenesis and support the robustness of myofibers. On the other hand, IL-6 has been recognized as a pro-inflammatory factor with pro-fibrotic actions. Elevated levels of IL-6 can deeply alter skeletal muscle milieu, affecting the activity and quality of cellular interactors in muscle regeneration and contributing to the fibrotic maladaptive response to tissue damage. Although a wealth of studies highlighted even more mechanisms regulating muscle regeneration/fibrosis, a comprehensive understanding of the complex interplay between the main cellular interactors in muscle niche is still lacking. Thus, future studies are required to develop specific therapeutic strategies in order to restore the physiologic relationship among cell mediators of muscle regeneration, limiting the extent of fibrosis.
